# 3D Micro-Expression Recognition Based on Adaptive Dynamic Vision

**DOI:** 10.3390/s25103175

**Published:** 2025-05-18

**Authors:** Weiyi Kong, Zhisheng You, Xuebin Lv

**Affiliations:** 1 National Key Laboratory of Fundamental Science on Synthetic Vision, Sichuan University, Chengdu 610065, China; 2021326040005@stu.scu.edu.cn (W.K.); youzs_scu_edu@163.com (Z.Y.); 2 College of Computer Science, Sichuan University, Chengdu 610065, China

**Keywords:** deep learning, intelligent perception, micro-expression recognition, emotion classification

## Abstract

In the research on intelligent perception, dynamic emotion recognition has been the focus in recent years. Small samples and unbalanced data are the main reasons for the low recognition accuracy of current technologies. Inspired by circular convolution networks, this paper innovatively proposes an adaptive dynamic micro-expression recognition algorithm based on self-supervised learning, namely MADV-Net. Firstly, a basic model is pre-trained with accurate tag data, and then an efficient facial motion encoder is used to embed facial coding unit tags. Finally, a cascaded pyramid structure is constructed by the multi-level adaptive dynamic encoder, and the multi-level head perceptron is used as the input into the classification loss function to calculate facial micro-motion features in the dynamic video stream. In this study, a large number of experiments were carried out on the open-source datasets SMIC, CASME-II, CAS(ME)^2,^ and SAMM. Compared with the 13 mainstream SOTA methods, the average recognition accuracy of MADV-Net is 72.87%, 89.94%, 83.32% and 89.53%, respectively. The stable generalization ability of this method is proven, providing a new research paradigm for automatic emotion recognition.

## 1. Introduction

In intelligent perception based on deep learning, technology for the perception of human emotions is currently a key research direction. The change in facial expression is an effective medium for relating internal emotional state, which can predict a person’s emotional state.

Facial expressions can be divided into macro-expressions (MaEs) and micro-expressions (ME) [1]. MaEs, also known as regular facial expressions, generally last for 0.5 s–4 s, and their intensity is generally high. MEs generally occur within 0.5 s, and their intensity is low. The occurrence of an ME is often accompanied by emotions such as stress and anxiety. Therefore, the research on intelligent perception of MEs is put forward emphatically, namely involving automatic facial expression recognition (FER). FER is widely used in education, medical care, national security, and entertainment [2]. According to different types of input data, it can be divided into static facial expression recognition (SFER) and dynamic facial expression recognition (DFER) [3,4]. SFER uses images as the input format, while DFER uses dynamic or video streams as the input format. Most SEFR research focuses on the recognition of MaEs, and this paper pays more attention to the detailed recognition of MaEs using DFER.

Researchers have developed a paradigm for DFER based on circular convolution neural networks. References [5,6,7] reported 2D and 3D deep convolution networks, recursive neural network models, as well as advanced transformer architectures. Although current DFER research has succeeded, issues such as a small number of data samples and the uneven distribution of categories lead to over-fitting of the model and poor generalization performance in the training process. Moreover, because MEs change too fast, the feature extraction module of the model needs high performance to effectively capture facial micro-motion information [8,9]. All of these have come to represent complex problems in DFER research.

In order to solve the above problems, simple and effective methods include increasing the number of data samples and intelligent generation. The advantage of this method is that it ensures the stability and generalization ability of the model, but the data samples produced by intelligent generation are only partially accurate. This is highly dependent on the performance of the generated model, which introduces some errors in the final recognition capability of DFER [10]. Another line of research is based on amplification methods, such as the classical Euler amplification method. The Euler amplification model is directly trained on continuous frames of images or video stream data, and ultimately the local amplification result is obtained to identify micro-expressions [11]. This method has been proven to be effective in many DFER models. However, there is a decline in the accuracy of emotion recognition caused by overly distorted local facial textures. Moreover, it cannot be used in real-time emotion prediction tasks.

In order to solve the above problems, we put forward two core designs comprising MADV-Net. An effective hierarchical adaptive dynamic feature extraction encoder is developed to solve the problems of small datasets and unbalanced data. Unlike the previous circular convolution’s global attention, the adaptive 3D partition feature extraction module proposed in this paper uses local and global feature information changes as much as possible by using fine-grained feature extraction and analysis in the texture layer, depth layer, and action unit (AU) encoders. Then, the micro-expression recognition algorithm of dynamic attention (MADV-Net) with fine-grained labeling for facial motion coding is studied, integrating the dual channels of AU coding and video coding. Specifically, in addition to using the inter-frame difference signals of texture and depth as auxiliary learning features, explicit temporal facial motion estimation is carried out simultaneously. In order to verify the effectiveness of MADV-Net, the SMIC dataset [12] is pre-trained, and then the pre-trained model is fine-tuned on the CASME-II [13], CAS(ME)^2^ [14], and SAMM datasets [15]. The results show that MADV-Net is significantly superior to other SOTA methods, which indicates that it can learn enough facial micro-motion information and perform reliable expression recognition.

The main contributions of this paper are summarized as follows:

(1) We propose a novel deep learning DFER research paradigm, MADV-Net, for macro–micro-expression mixed data. By leveraging self-supervised fine-tuning models to learn sufficient global features, combined with fine-grained local feature learning strategies, we design a DFER paradigm model with high reliability and generalization capabilities.
(2) The video stream data are divided into low-dimensional, middle-dimensional, and high-dimensional feature streams through the design of adaptive and dynamic partition feature extraction models, and the information on inter-frame differences is learned through dynamic adaptive factors. Finally, the pyramid structure fuses feature information streams for subsequent fine-grained recognition and classification.
(3) The dual-channel attention model of AU coding and the image and video encoder are innovatively designed, and adaptive dynamic adjustment of feature information is skillfully used. The multi-level features are dynamically fused and output to the multi-level head perceptron. Then, a fine-grained classification function is used to accurately identify the emotions in the video stream in real time.

## 2. Related Work

### 2.1. Expression Recognition Algorithm Based on Visual Encoder

The main body of the visual transformer (ViT) model is the Encoder part based on the Transformer model, as described in many related studies [16]. From 2019 to 2025, many excellent studies were published on image and video recognition and classification [17,18]. Among them, the most significant one was put forward by Nagarajan, P. and other scholars in 2022, mainly used for image classification [19]. Its architecture design involves dividing the image into 16 small blocks, inputting the small blocks into the embedded layer of a linear projection of a plane small block, and then obtaining vectors, which are called marks. Then, a series of labels are preceded by new category tags. In addition, the position information is needed, which is then input into the transform encoder. The input and output of the transform encoder are in one-to-one correspondence, and finally, the information is classified.

On this basis, researchers such as Arnab, A. put forward a ViT (ViViT) for feature extraction from video data, which mainly studies video formats containing time-series information dimension [20]. In order to efficiently deal with the large-scale spatiotemporal signs generated in video data, they proposed and discussed several approaches for decomposing the spatial and temporal dimensions and then proposed the corresponding network architecture to increase the efficiency and scalability of the model for feature extraction from video data [21,22]. Then, the model’s training is standardized and tested on a small dataset, which shows promising results. It adopts a video embedding method, which differs from traditional two-dimensional image data. Video data are equivalent to sampling in three-dimensional space (adding a time dimension). The approaches mentioned in ViViT are all obtained by mapping the video data to the logo and then adding position coding to transpose the logo to obtain the final input. Then, a network model of spatiotemporal attention is designed through uniform sampling and spatiotemporal pipeline sampling. This method is relatively simple, but the main problem is that it will introduce an exponentially increasing amount of calculation, resulting in low efficiency. Therefore, in [23], the model two-factor decomposition encoder network is improved, and this model carries out a relatively independent processing approach for space and time, respectively. Firstly, the spatial encoder models the symbol with the same time index, and cls_token is the output. After that, the output category mark and the frame dimension representation mark are spliced and input into the time encoder to obtain the final result.

These studies have made significant contributions to image and video data recognition and classification based on deep learning, but there are still some issues. The fundamental ViT model requires massive amounts of data for pre-training, and whether it demonstrates good generalization capabilities for heterogeneous datasets warrants further exploration [24]. The stacked module design of the network makes it difficult to guarantee the algorithm’s running speed, requiring robust and expensive GPU support for research.

### 2.2. Micro-Expression Recognition Method Based on AU Coding

In psychology, a model describes expressions by coding facial muscle movements, known as the “Facial Action Coding System (FACS)” [25]. FACS contains a set of units used to encode specific facial muscle movements, called action units (AUs) [26]. The correspondence between facial muscle changes and different expressions was summarized by the renowned psychologists Paul Ekman and W.V. Freeson in 1976 through observation combined with biofeedback. Based on anatomical characteristics, it was concluded that there are 42 facial muscles controlled by different brain regions. Some can be directly controlled consciously (voluntary muscles), while others are not easily consciously controlled (involuntary muscles) [27]. Detailed information about the AUs is shown in Table 1.

Recently, in expression recognition research based on AU coding, ref. [29] proposed a baseline method using a classical convolutional neural network model combined with graph volume product-designed AU coding to realize micro-expression recognition in video streams. Additionally, the studies in [30] demonstrate that preprocessing datasets before feature analysis can improve emotion recognition accuracy. However, preprocessing approaches such as virtual data generation, emotional approaches, and filters may introduce miscellaneous information and increase computational parameters [31].

In addition, the method of combining region of interest and AU coding to detect MEs is proposed in [32]. As a basic muscle unit, an AU is coded as a feature, and through the design of 3DCNN, a feature recognition model of the texture layer is formed in cascade.

Currently, the accuracy of expression recognition using AU coding is 50% to 78% [33,34,35]. The rapid occurrence and disappearance of micro-expressions and the imbalance of samples between classes make it difficult to improve the accuracy of this kind of research.

With the deepening of the research on automatic ME recognition based on an efficient deep learning model, a reliable rapid feature extraction model is needed to realize a real-time micro-expression recognition system. It is necessary to train a micro-expression recognition algorithm that can be applied to video coding by studying the effective use of AUs and combining them with a high-performance depth model.

## 3. Proposed Method

For such few-shot learning tasks, visual encoders have difficulty achieving ideal results in video recognition when they are not sufficiently pre-trained. This is due to the overfitting phenomenon caused by the powerful modeling ability of Transformer and the lack of inductive bias. To address the above issues, an effective approach involves controlling the model capacity and enhancing its scalability, thereby reducing the number of parameters while improving performance. This paper adopts the idea of transfer learning and transfers the knowledge in the macro-expression recognition task to the micro-expression recognition task. Firstly, the powerful and efficient Depth Anything [36] open-source architecture is used to obtain the corresponding 3D video. 3D video refers to video data containing depth information (z-coordinate) for each pixel (x, y). The depth represents the distance of scene points from the camera (or reference point), encoding 3D geometry and 2D visual information of the scene. Unlike “volume video” (voxel grid with x, y, z coordinates), the “3D video” discussed herein is a 2D video with per-pixel depth (depth map). Each frame consists of x, y pixel dimensions and a z depth channel, forming a 3D representation of scene geometry. Subsequently, we enhance the performance of capturing local features of dynamic micro-expressions through the design of a dynamic decoding model and the introduction of a 3D visual encoding module. Simultaneously, by integrating AU encoding features, we extract the dynamic characteristics of facial units, effectively addressing the challenge of subtle local feature variations. These variations arise from the low intensity of facial muscle movements during micro-expression recognition, ensuring a more accurate and comprehensive analysis of micro-expressions. Finally, more accurate recognition of dynamic micro-expressions is achieved by learning the fine-grained features of errors. The following will elaborate on this algorithm in detail.

### 3.1. Multi-Level Adaptive Dynamic Visual Attention Network Model Design

Due to the short duration of micro-expressions, it is difficult to capture large-scale data. Meanwhile, the muscle shape of micro-expressions becomes weak, which causes difficulties in improving recognition accuracy. Moreover, previous Transformer-based methods require a lot of training data and computing resources. These problems all limit the further exploration of small-sample class dynamics problems.

In response to the above challenges, this paper innovatively proposes a three-dimensional convolutional Transformer network architecture—the multi-level adaptive dynamic visual attention network model (MADV-Net)—for fine-grained micro-expression recognition. The model combines the advantages of 3D-CNN and Transformer and fully considers the time dimension, spatial dimension, and AU facial information in the video, to overcome the limitations of the existing methods in dealing with the dynamic problems of small samples and improve the accuracy of micro-expression recognition.

**Figure 1:**
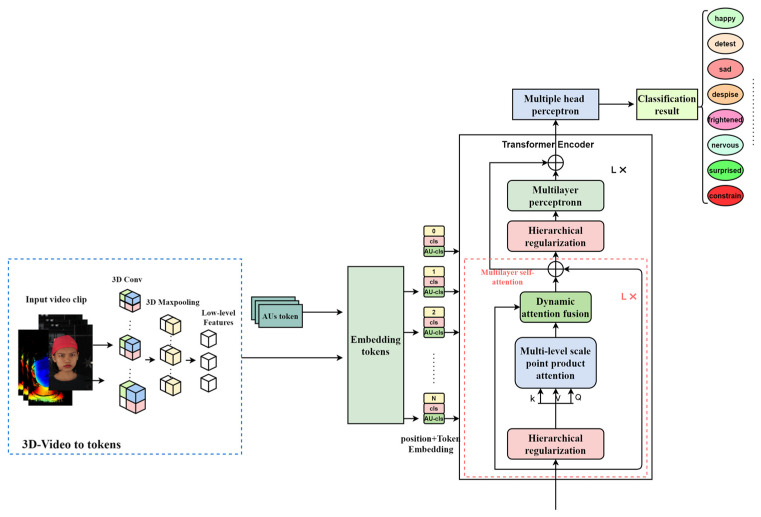
General architecture of MADV-Net.

MADV-Net innovatively integrates the texture path, video 3D depth image path, and AU coding path of micro-expression video, with the two-dimensional texture video flow, the prediction depth video stream, and the AU coding flow as inputs. The multi-level self-attention mechanism proposed in this paper effectively integrates local and global feature information through multi-scale feature interaction and adaptively focuses the pixel areas with high attention weights, so as to build a dynamic high-dimensional feature representation. Combined with the dynamic fusion module, this method embeds the most discriminative features into the emotional feature extraction process, strengthens the feature learning of high-attention sub-blocks, and significantly improves the discrimination ability of high-dimensional features of micro-expressions. Finally, the accurate recognition of micro-expressions is realized by integrating the facial action unit (AU) coding features with the local features of dynamic visual attention. The architecture of MADV-Net is shown in Figure 1.

### 3.2. Video Feature Encoding Based on Adaptive Dynamic Regulation

In the deep learning training process, serious overfitting will occur due to the small sample size of the micro-expression dataset and the imbalance of the data distribution. Therefore, it is particularly important to enhance the extraction and analysis of intra-class features for the identification of micro-expression video streams. A detailed analysis of the deep convolutional mapping in MADV-Net, the AU feature coding sub-module-based attention modules, and the adaptive modulation is presented below.

#### 3.2.1. Deep Convolution Mapping

**Figure 2:**
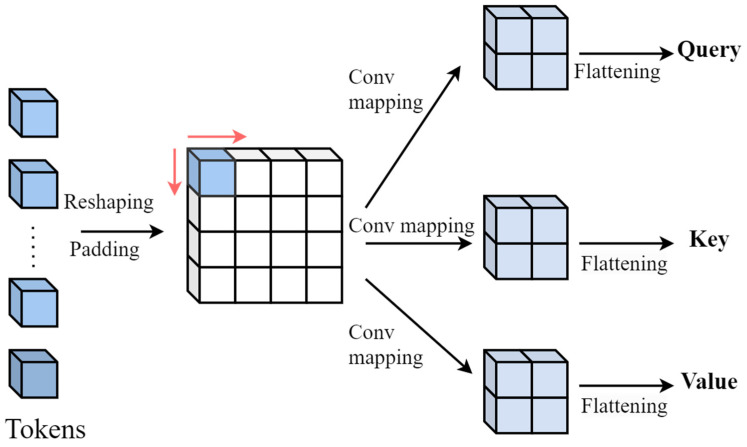
Deep convolution mapping.

The video-based micro-expression recognition method extracts features from the spatial and temporal dimensions of the input video. In order to combine the long-distance relationship modeling ability of Transformer with the advantages of CNN low-frequency feature extraction, this section proposes a multi-head self-attention deep convolution mapping method. On the one hand, the architecture of layer-by-layer deep convolution mapping is adopted to generate query (Q), key (K), and value (V) embedding. On the other hand, considering cost savings and input marking, a convolutional design is introduced into Transformer to realize multi-head self-attention, thus replacing the original linear mapping based on a full connection layer. The specific operation is shown in Figure 2. The input tokens are reconstructed into a token graph, and then the separable convolution layer is used as the convolution map, with the convolution kernel size being s×s.

In depth convolution mapping, two kinds of filters are needed for convolution operation. Firstly, an independent s×s filter is used for each channel to generate the feature map, and then a 1×1 filter is used to obtain the point-by-point linear combination of the feature map. After that, the tokens are flattened and projected into the initial dimension. Finally, the output data are normalized in batches. The calculation formula is as follows in Equation (1):

(1) zlq,k,v=Flatten(Conv2d(Reshaped2d(zl),s)),

where $z_{l}$ represents tokens before convolution projection and $z_{l}^{q,k,v}$ represents token inputs of the query (Q), key (K), and value (V) matrices of the first layer.

In neural network architectures, the Flatten operation is applicable to scenarios where a transition from multi-dimensional tensors (such as 2D or 3D feature maps) to 1D vectors is required. From a mathematical perspective, consider a tensor T with a shape of (a,b,c,...), where a,b,c,... represent the respective dimensions of the tensor. The flattening operation transforms this tensor into a 1D vector, whose length is the product of all dimensions of the original tensor, i.e., a,b,c,.... In Equation (1), the Flatten operation acts on the output of a 2D convolution (Conv2d) (i.e., a multi-dimensional tensor) to convert it into a 1D vector, thereby enabling it to serve as input for subsequent operations in the neural network, such as being fed into a fully connected layer.

Conv2d adopts a deeply separable convolution structure, which consists of three continuous operations: depthwise convolution, batch normalization, and pointwise convolution. Among these, the convolution kernel size of depth convolution is set in the experiment. This design reduces the computational complexity while maintaining feature representation ability through point-by-point fusion of depth feature extraction of the spatial dimension and channel dimension. The batch normalization operation is used to standardize the distribution of activation values and stabilize the training process.

- 3D-video-to-tokens module

The neighboring pixels between frames in micro-expression videos have a strong correlation. Therefore, modeling the relationship between neighborhood features in video frames is very important for the fine-grained recognition of micro-expressions. However, the traditional Transformer cannot make full use of the prior information in the video, and directly marking tokens, which is a large video processing task, will lead to difficulty in training and the loss of low-frequency features. In order to make full use of the advantages of Transformer’s dynamic attention and weight sharing in CNN, this section introduces the 3D-video-to-tokens module of MADV-Net in detail.

Given a video clip $V\in R^{T\times H\times W\times C\times D}$, where T is the video length, H is the video frame width, W is the video frame height, C is the number of channels, and D is the depth, the 3D-video-to-tokens module can be expressed as Equation (2):

(2) $$Out(Z^{\prime })=3D\_Maxpool(BN(3D-Conv(z))),$$

where z represents the token tensor of the input video segment, $z^{\prime }\in R^{T^{\prime }\times H^{\prime }\times {\dot{W}}^{\prime }\times C^{\prime }\times D^{\prime }}$ represents the output tokens of the video to the marking module, and $Out(z^{\prime })$ represents the output of the module—that is, the extracted tokens.

Because the feature matrix is a three-dimensional matrix, the embedded filter is represented by a three-dimensional tensor. This design makes full use of the advantages of the CNN model and can extract low-frequency features and establish the relationship between neighboring pixels in the video.

#### 3.2.2. AU-Based Feature Coding Submodule

Firstly, according to the definition and description of facial motion coding units, the difference in AU intensity between human face- and micro-expression-driven change is defined as $V\overset{\Delta }{=}V_{x}-V_{t}$, where $V_{x}$ represents the AU vector of the micro-expression deformation frame and $V_{t}$ represents the AU vector of the static face. Each AU represents the intensity value of motion change in the corresponding area, and c indicates there is a category c emotion. Then, the whole network can be defined and expressed as Equation (3):

(3) $$v_{0}^{c},v_{1}^{c},\ldots ,v_{n-1}^{c},{\tilde{y}}^{c}=F_{0}(x^{c}),F_{1}(F_{0}(x^{c})),\ldots ,F_{n}(F_{n-1}(...F_{0}(x^{c})\ldots )),$$

where $F_{i}$ represents feature transformation functions/layers in the network. Each $F_{i}$ maps the input feature from the previous layer ($F_{0}(x^{c})$ extracts initial features from the input, $x^{c},F_{1}(F_{0}(x^{c}))$ transforms these features), building a hierarchical feature representation.

Then, we define the information bottleneck as shown in Equation (4):

(4) $$\eta_{IB}=\beta \eta (x^{c};v_{n-1}^{c})-\eta (v_{n-1}^{c};y^{c}),$$

where β represents scaling hyperparameter and η represents information metric.

According to the variational upper bound formula, the above formula is expressed as Equation (5):

(5) $${\tilde{\eta }}_{IB}=E_{x^{c}\sim p(x^{c})}[\beta KL[P(v_{n-1}^{c}|x^{c})||Q(r_{n-1}^{c})]-E_{v_{n-1}^{c}\sim p(v_{n-1}^{c}|x^{c})}[\log Q(y^{c}|v_{n-1}^{c})]],$$

where E represents the expectation operator, averaging over the distribution of $x^{c}\sim p(x^{c})$. KL represents Kullback–Leibler divergence, measuring the difference between two distributions ($P({\tilde{v}}_{n-1}^{c}|x^{c})$ and $Q({\tilde{v}}_{n-1}^{c})$). P represents a true probability distribution. Q represents variational distribution (approximate posterior), used to simplify inference.

Each layer of the network model in this section uses similar monitoring objectives, which are expressed in Equation (6):

(6) $$\eta_{i}^{c}=\beta \cdot \eta (v_{i-1}^{c};{\tilde{v}}_{i}^{c})-\eta (v_{i}^{c};{\tilde{v}}_{i}^{c}),$$

where ~ is the representation of this layer after extracting information—that is, the weight given by the model according to the degree of judging the participation of this layer’s characteristics in the calculation.

According to the variational upper bound formula, Equations (5) and (6) can be transformed into Equation (7):

(7) $${\tilde{\eta }}_{i}^{c}=E_{v_{i-1}^{c}\sim p(v_{i-1}^{c})\left\lfloor \beta KL[P({\tilde{v}}_{i}^{c}|v_{i-1}^{c})||Q({\tilde{v}}_{i}^{c})]-E_{v_{i}^{c}\sim p(v_{i}^{c}|v_{i-1}^{c})}[\log Q({\tilde{v}}_{i}^{c}|v_{i}^{c})\right\rfloor },$$

where the lower index of E specifies the distribution over which the expectation is taken. In (7), the first term represents the expectation of the feature distribution under the condition of input $x^{c}$ (${\tilde{v}}_{i}^{c}\sim p({\tilde{v}}_{i}^{c}|x^{c})$). The second term represents the expectation of the output distribution under the characteristic condition of layer i (${\tilde{v}}^{c}\sim p({\tilde{v}}^{c}|{\tilde{v}}_{i}^{c})$).

From the above deduction, it can be seen that the learning mean and variance of the model are not related to each other. Therefore, in order to simplify the calculation, only the mean value is optimized in the method. Therefore, the content irrelevant to the mean can be simplified, and the simplified formula is given in Equation (8):

(8) $${\tilde{v}}_{i}^{c}=\beta \cdot {\left(v_{i}^{c}\cdot \mu_{i}^{c}\right)}^{2}+||v_{i}^{c}-\mu_{i}^{c}\cdot r_{i}^{c}|{|}_{2}^{2},$$

For the classifier at the last level, the objective is to maximize the mutual information between the extracted classification and labels, and the expression formula is Equation (9).

(9) $$\eta_{n}^{c}=-\eta ({\tilde{y}}^{c},y^{c}),$$

The corresponding lower bound of variation is expressed in Equation (10):

(10) $${\tilde{\eta }}_{n}^{c}=E_{v_{n-1}^{c}\sim p(v_{n-1}^{c})}[E_{{\tilde{y}}^{c}\sim p({\tilde{y}}^{c}|v_{n-1}^{c})}[-\log Q(y^{c}|{\tilde{y}}^{c})]],$$

Then, assume that Q is a label with polynomial distribution, and the loss is expressed as Equation (11):

(11) $$\begin{array}{l} {\tilde{\eta }}_{n}^{c}=-y^{c}\log f_{n}(v_{n-1}^{c})-(1-y^{c})\log(1-f_{n}(v_{n-1}^{c}))\\ \begin{array}{c} \;\;\;=-y^{c}\log{\tilde{y}}^{c}-(1-y^{c})\log(1-{\tilde{y}}^{c}) \end{array} \end{array},$$

Therefore, the overall loss function $L_{AU}$ of the final AU feature encoder is the sum of all layer losses, as shown in Equation (12):

(12) $$L_{AU}={\tilde{\eta }}^{c}=E_{x^{c}\in X^{c}}[\sum_{i=0}^{n}{\tilde{\eta }}_{i}^{c}],$$

#### 3.2.3. Adaptive Attention Adjustment

Inspired by the Transformer architecture, the attention structure of time and space is improved, the time and space dimensions of input micro-expression video data are decomposed at multiple levels, and the global and local feature change information is effectively used, so as to learn the feature change in micro-expression in real time, aiming at recognizing micro-expression with high precision and high speed.

The facial muscle deformation intensity of micro-expression is low, which indicates that only a small area of the image will be affected, which requires multi-head self-attention (MSA) to focus on the changing area. To make the model converge faster, the following three kinds of attention are used to pay more attention to the blocks with higher weights and discard the blocks with lower attention weights in the training process to learn the deformed features faster.

The structure of MADV-Transformer encoder in this section is composed of the feature vector $f_{p\times q}$ created by the 3D embedded model and the classification model of classification mark $f_{c,q}$. Then, the position relationship of each patch is expressed by using the learnable position embedding $f_{pos}$, and it is input into the MADV-Transformer for training through weighted fusion to generate a new feature $f_{(p+1)\times q}$.

The new feature vector is expressed as Equation (13):

(13) $$f_{(l+1)\times q}=[f_{c,q},f_{1,q},f_{2,q},...,f_{p,q}]+f_{pos},$$

where p represents the number of image blocks and $f_{i,q}$ represents the embedded projection of image blocks.

The attention weight of each image block is learned by using this category label. MADV-Transformer needs to encode the position of image blocks, use learnable position embedding $f_{pos}$ to represent the position relationship of each image block, input it into MADV-Transformer model, and finally train it through the weighted fusion function. The classification process is represented by Equations (14)–(17):

(14) $$y^{\prime }=soft\max(F_{LR}\left\|f_{Att},f_{Conv}\right\|),$$

(15) $$f_{Att}=F_{MLP}(f_{N}),$$

(16) $$f_{i}=F_{MSA,i}(F_{MSA,i}(f_{i-1})),i=1,2,...,L,$$

(17) $$f_{Conv}=F_{Conv}(f_{p\times q}),$$

where $y^{\prime }$ represents the category of the identified micro-expression, $F_{LR}$ represents the fully connected layer, $f_{(l+1)\times q}$ represents the attention feature output by the MADV-Transformer encoder, N represents the number of modules, $F_{MSA,i}$ and $F_{MLP,i}$ represent the i-th layer modules, and $F_{C\mathrm{onv}}$ represents a convolution layer.

#### 3.2.4. Loss Function

In the MDAV-Net model training, the joint optimization loss function is used to optimize the model, which combines the multi-head classification loss function, multi-frequency loss function, and the above-mentioned AU loss. The overall loss function is defined in Equation (18):

(18) $$L=L_{Multi-class}+L_{freq}+L_{AU},$$

where the calculation formula of the multi-head classification loss function $L_{Multi-class}$ is given in Equation (19):

(19) $$L_{Multi-class}=-\mu_{i}{\left(1-p_{i}\right)}^{\lambda }\cdot \log(p_{i}),$$

where μ is a balance factor and λ indicates the decreasing rate of adjusting the weight of simple samples. μ and λ are both hyperparameters.

For Equation (18), $L_{freq}$ represents the multi-frequency loss function, and its composition is as shown in Equation (20):

(20) $$L_{\mathrm{freq}}=\alpha L_{low}+\beta L_{mid}+\gamma L_{high},$$

where α,β,γ are the weight coefficients used to balance the classification loss of different frequency features. $L_{low},L_{mid},L_{high}$ represent the loss functions of low frequency, intermediate frequency, and high frequency, respectively. The low-frequency classification loss is expressed as Equation (21):

(21) $$L_{low}=-\sum_{i=1}^{C}y_{i}\log(p_{low,i}),$$

where c represents the number of categories, $y_{i}$ is the one-shot code of the real tag, and $p_{low,i}$ is the probability of the model predicting the low-frequency features.

The classification loss of intermediate frequency features is expressed as Equation (22):

(22) $$L_{mid}=-\sum_{i=1}^{C}y_{i}\log(p_{mid,i}),$$

where $p_{mid,i}$ is the class i probability of the model’s prediction of intermediate frequency characteristics.

The classification loss of high-frequency features is expressed as Equation (23):

(23) $$L_{high}=-\sum_{i=1}^{C}y_{i}\log(p_{high,i}),$$

where $p_{high,i}$ is the class i probability of the model’s prediction of intermediate frequency characteristics.

#### 3.2.5. Algorithm Complexity

Firstly, the computational complexity of 3D convolution is discussed. Let the input features be $T\times H\times W\times C_{in}$ (T is the time dimension, H,W are the height and width of the space, respectively, and $C_{in}$ is the number of input channels), the convolution kernel size be $K_{t}\times K_{h}\times K_{w}$, and the number of output channels be $C_{out}$. The multiplication number of a single 3D convolution operation is $K_{t}\times K_{h}\times K_{w}\times C_{in}$, and the total multiplication number is $T\times H\times W\times C_{out}\times K_{t}\times K_{h}\times K_{w}\times C_{in}$ when calculating the whole input feature. Then, the time complexity is $O(THWC_{out}K_{t}K_{h}K_{w}C_{in})$.

Three-dimensional maximum pooling is mainly a traversal operation. Let the size of the pooled kernel be $S_{t}\times S_{h}\times S_{w}$, and when the input feature T×H×W×C is pooled, each element needs to traverse the size of the elements in the pooled kernel, and the time complexity is O(THWC).

For a single MDAV-Transformer layer, the total complexity is the sum of the adaptive multi-head attention and MLP complexity, so the complexity of a single MDAV-Transformer layer can be expressed as follows (24):

(24) $$O(3Ld^{2}+L^{2}d+2Ldd_{hidden}),$$

For an n-layer MDAV-Transformer, its total complexity can be expressed as (25):

(25) $$O(N(3Ld^{2}+L^{2}d+2Ldd_{hidden})),$$

To sum up, assuming that the output features of the 3D convolution module are processed to obtain the input of the MDAV-Transformer, the total time complexity of the model is $O(THWC_{in}C_{out}K_{t}K_{h}K_{w})+O(N(3Ld^{2}+L^{2}d+2Ldd_{hidden}))$.

Next, the analysis and comparison of the experimental dataset and experimental results, the ablation experiment, and the visual analysis of the model structure will be introduced in detail. In this study, the open-source facial micro-expression datasets CASME-II [13], CAS(ME)^2^ [14], SMIC [12], and SAMM [15] were systematically tested and analyzed, and the generalization of the model was verified based on the AU data sample. The results fully proved the adaptability and robustness of the model in cross-dataset scenarios.

## 4. Experimental Setup and Experimental Results

### 4.1. Introduction of Dataset

The experiments in this section were fully performed using the SMIC [12], CASME-II [13], CAS(ME)^2^ [14], and SAMM [15] datasets.

SMIC [12] contains three subsets, namely SMIC-HS, SMIC-VIS, and SMIC-NIS, among which SMIC-HS has the largest sample size and frame rate, and most of the research on expression and micro-expression recognition uses SMIC-HS. Therefore, this section also uses these data subsets for the experiments, consisting of 164 samples from 16 subjects and containing three major types of emotions, namely surprised, positive, and negative.

CASME-II [13] consists of 255 video samples, including 26 subjects. At first, the collected emotions included seven categories, namely happiness, disgust, depression, surprise, sadness, fear, and others. However, after the real emotions were collected, the samples of sadness and fear among the subjects were found to be too small, so the samples of these two categories were deleted, and the final effective emotions were separated into five categories, namely happiness, disgust, depression, surprise, and others. In the experiment in this section, the video data were first divided into corresponding image datasets frame by frame, which were used as the experimental inputs for the experiment.

The CAS(ME)^2^ [14] dataset is a high-quality resource in the field of micro-expression research. It contains data from various participants in different experiments and has been strictly manually labeled and verified, including happiness, sadness, surprise, fear, disgust, anger, contempt, and others, totaling eight types of micro-expression data. It covers multi-modal data sources, and besides the micro-expression video, it also collects synchronous physiological signals, such as skin electricity and heart rate, which can fully reflect the individual’s physical and mental state when the micro-expression is generated. In addition, the data from CAS(ME)^2^ re marked in detail, including the time information and intensity grade of expressions, which provides a reliable data basis for the study of micro-expressions.

SAMM [15] consists of 32 subjects and 159 samples, including eight emotional samples, namely happiness, surprise, contempt, anger, others, disgust, fear, and sadness. Some studies think that the number of samples representing three of these emotions is too low, so they discard these data and study the classification and recognition results for the other five emotions. However, in order to verify the effectiveness of the fine-grained module, the research in this section retained the corresponding data of these three emotions.

### 4.2. Evaluation Index

For common binary classification problems, the F1-score is often used to evaluate the classification performance, while for multi-classification problems, the macro-F1 score is usually used. Its advantage is that it treats each category equally and is not affected by the number of category samples.

First, we calculated the accuracy and recall of each category. For each category, the calculation formulas are given in Equations (26) and (27):

(26) $$precision_{i}=\frac{TP_{i}}{TP_{i}+FP_{i}},$$

(27) $$Recall=\frac{TP_{i}}{TP_{i}+FN_{i}},$$

where $TP_{i}$ represents the real example of the category i, $FP_{i}$ represents the false positive example of the category i, and $FN_{i}$ represents the false negative example of the category i.

Then, the accuracy and recall of all categories are averaged to obtain the macro-precision and macro-recall, and the calculation formulas are given in Equations (28) and (29), respectively.

(28) $$Macro-precision=\frac{1}{N}\sum_{i=1}^{N}precision_{i},$$

(29) $$Macro-Recall=\frac{1}{N}\sum_{i=1}^{N}Recall_{i},$$

where N represents the category.

Finally, macro-precision and macro-recall are used to calculate macro-F1, as shown in Equation (30):

(30) $$M\mathrm{acro}-F1=\frac{2\times (M\mathrm{acro}-precision)\times (M\mathrm{acro}-Recall)}{\left(M\mathrm{acro}-precision)+(M\mathrm{acro}-Recall\right)},$$

The UF1 (unweighted F1-score) and UAR (unweighted average recall) evaluation indicators are introduced in detail below.

UF1 is an unweighted F1 score, which is calculated by calculating the F1 score of each category separately and then taking the average value. The F1 score is the harmonic average of precision and recall, which is used to measure the comprehensive performance of the model in each category. The formula is shown in Equation (31).

(31) $$UF1=\frac{1}{C}\sum_{c=1}^{C}\frac{2\times \sum_{i=1}^{k}TP_{c}^{i}}{2\times \sum_{i=1}^{k}TP_{c}^{i}+\sum_{i=1}^{k}FP_{c}^{i}+\sum_{i}^{k}FN_{c}^{i}},$$

where C is the total number of micro-expression categories, k is the number of folds of cross-validation (for one-way cross-validation, k is equal to the number of samples), and $TP_{c}^{i}$ represents the number of true positives of category c in the i-fold cross-validation. $FP_{c}^{i}$ represents the number of false positives of category c in the i-fold cross-validation. $FN_{c}^{i}$ indicates the number of false negatives of category c in the i-fold cross-validation.

UAR refers to the unweighted average recall rate, which is calculated by calculating the recall rate of each category separately and then taking the average. The recall rate measures the proportion of positive cases correctly identified by the model in each category. UAR is expressed as shown in Equation (32).

(32) $$UAR=\frac{1}{C}\sum_{c=1}^{C}Acc_{c},$$

where $Acc_{c}=\frac{TP_{c}}{n_{c}}$, C is the total number of micro-expression categories, $Acc_{c}$ is the accuracy of category c, and $TP_{c}$ is the total number of real cases of category c—that is, the sum of real cases of category c in cross-validation of all folds. $n_{c}$ is the total number of samples in category c.

### 4.3. Contrast Experiment

To demonstrate the effectiveness of the MADV-Net method proposed in this paper, 13 advanced methods in the field of micro-expression recognition were compared. These included C3D [37] based on 3D convolutional networks, R(2 + 1)D-18 [38], 3D ResNet-18 [39] and EC-STFL [40]. We also included models designed by combining the Resnet series of networks with the LSTM architecture based on recurrent neural networks (Resnet-18 + LSTM [41] and Resnet-18 + GRU [42]), improved networks of the Transformer architecture (Former-DFER [43], CEFLNet [44], EST [45], and STT [46]), and variants of dynamic vision models (NR-DFERNet [47], VideoMAE [48], and MAE-DFER [49]). These advanced methods enhance the accuracy of micro-expression recognition by fusing 3D feature information and improve the feature analysis of micro-expressions by combining methods for dynamically capturing consecutive-frame videos. Therefore, representative studies were selected for each different model design as comparative experiments to prove the advancement of the method proposed in this paper.

The model was trained and predicted on GeForce RTX 3090, CUDA 11.6, and PyTorch 2.0.1, and the performance of the model in four micro-expression datasets was verified. Among them, the batch size was 64, the optimizer adopted an AdamW learning rate set to 5 × 10^−5^, the weight attenuation was 0.05, the learning rate adopted CosineAnnealingLR with the maximum round of 300 and the minimum learning rate, and FP16 was used to accelerate the calculation with semi-precision to reduce the memory occupation.

#### 4.3.1. Experimental Results on SMIC Dataset

In the following comparative experiments, all algorithms performed emotion recognition based on video streams, which ensured the fairness of these experiments. As can be seen from Table 2, based on the SMIC [12] dataset, the recognition accuracy of all algorithms for positive emotions is higher than that for negative and surprised emotions. Analysis shows that the model design of convolutional networks is more sensitive to capturing positive emotions. This is because when positive emotions occur, the facial texture changes to a larger extent, enabling convolutional networks to extract features with higher accuracy. Therefore, the correct recognition rate of positive emotions after final fusion is relatively high. Among them, the recognition accuracy of MADV-Net for positive samples reaches 84.31%, and the average recognition accuracy for the three emotions is 72.87%. The experimental results outperform those of the 13 mainstream algorithms. This proves that MADV-Net achieves good recognition results in the three-class classification recognition task of the SMIC [12] data samples. In addition, the results indicate that the macro-F1 value is positively correlated with the average recognition accuracy, suggesting that all algorithms perform stably on the SMIC [12] dataset. The macro-F1 value of the method presented in this section reaches 0.69, which is higher than that of the other methods, indicating that this algorithm has better stability.

#### 4.3.2. Experimental Results on CASME-II Dataset

In the experiment described in this section, MADV-Net was compared with the current mainstream SOTA (state-of-the-art) methods based on the micro-expression benchmark dataset CASME-II [13]. The single-class recognition accuracy, average recognition accuracy, and macro-F1 score of five target emotions were systematically recorded. The experimental results are shown in Table 3.

Through analysis, it can be seen that MADV-Net demonstrates significant advantages in multi-dimensional performance indicators. Firstly, it leads other methods in both single-class and overall recognition accuracy. In the category of happiness, MADV-Net achieved the highest single-class recognition accuracy of 94.12%, which is 6.3 percentage points higher than the second-best method. Its average recognition accuracy reached 89.94%, surpassing all the other compared algorithms. This result indicates that the algorithm has a particularly prominent ability to capture the dynamic features in micro-expressions. In particular, it can accurately model the subtle movement patterns of facial muscle groups (such as the zygomatic major muscle and the orbicularis oculi muscle) during positive emotions. Secondly, MADV-Net has the advantages of cross-category stability and balance. As can be seen from the macro-F1 score (0.84), which is a core indicator reflecting category balance, MADV-Net performs more evenly across various emotions, higher than the highest value (0.82) of the compared methods. This advantage stems from the algorithm’s in-depth integration of multi-modal features (spatial texture features and time-series dynamic features), which can effectively capture the dynamic change patterns with extremely short durations (about 150 ms on average) in micro-expression sequences, avoiding the category bias problem caused by the insufficient utilization of time-series features in traditional methods. Finally, the robustness of the algorithm was verified in complex scenarios. The CASME-II dataset [13] contains a large number of spontaneous micro-expression samples, which present challenges such as low expression intensity, large individual differences, and complex background noise. By combining the multi-resolution feature pyramid and the dynamic attention mechanism, MADV-Net can still accurately locate key regions (such as the mouth and eyebrows) in micro-expression frames with a low signal-to-noise ratio and suppress irrelevant noise interference. The experimental results show that its performance in the fast transient surprise emotion (with a recognition accuracy of 88.24%) is better than that of the SOTA methods, verifying the algorithm’s universality for complex micro-expressions.

#### 4.3.3. Experimental Results on CAS(ME)^2^ Dataset

MADV-Net achieved good recognition results for seven emotions based on the CAS(ME)^2^ [14] dataset, with the accuracy for happiness, sadness, neutrality, anger, surprise, contempt, and fear being 92.90%, 78.99%, 79.02%, 84.36%, 81.36%, 85.37%, and 81.25%, respectively. The results are shown in Table 4.

MADV-Net demonstrates excellent performance across all seven emotion categories. Notably, it achieves an impressive recognition accuracy of 92.90% for the happiness category, outperforming the second-best method by 8.7 percentage points. The overall average recognition accuracy reaches 83.32%, marking a significant improvement of 20.31% compared to the model integrating CNN and RCN and a 6.18% enhancement over the latest state-of-the-art (SOTA) method, MAE-DFER [48]. This superiority stems from the algorithm’s unique multi-scale feature fusion mechanism. By constructing a hierarchical feature pyramid, MADV-Net can not only capture the subtle texture changes in facial muscles in micro-expressions but also effectively integrate the dynamic temporal information of expression sequences, enabling precise modeling of the characteristic patterns of different emotion categories. The proposed method exhibits remarkable cross-category balance and robustness. With a macro-F1 score of 0.82, MADV-Net surpasses the CNN-RCN model by 0.18 and outperforms MAE-DFER by 0.06, highlighting its high level of consistency across various emotion categories. This characteristic is attributed to the innovative improvements made to the Transformer architecture. Through the dynamic attention mechanism, the model can adaptively focus on critical areas of expressions, such as the eye and mouth action units, while effectively suppressing noise interference caused by individual differences and pose variations. In the recognition of low-intensity emotions (e.g., the neutral category, with an accuracy of 79.02%) and rapid transient expressions (e.g., the surprise category, with an accuracy of 81.36%), MADV-Net maintains stable performance, validating its generalization ability in complex micro-expression scenarios. MADV-Net benefits from the synergistic effects of its architectural innovations. The algorithm’s outstanding performance is largely due to its hybrid architecture. On one hand, the convolutional module efficiently extracts local features from micro-expression images. On the other hand, the Transformer-based temporal modeling module analyzes long-range dependencies in expression sequences. These two components complement each other through a bidirectional feature fusion mechanism. This architectural design not only overcomes the problem of disjointed spatial and temporal information in traditional methods but also significantly enhances the model’s understanding of the dynamic evolution of micro-expressions through end-to-end training optimization, ultimately achieving a breakthrough in emotion recognition performance.

#### 4.3.4. Experimental Results on the SAMM Dataset

Based on the important benchmark dataset SAMM [15] for micro-expression research, this section conducts recognition tasks for eight target emotions. The experiment reveals that the recognition accuracies of three emotions, namely happiness (93.95%), sadness (88.85%), and despisal (90.06%), are significantly higher than those of other categories. This is closely related to the clear spatial distribution characteristics of the facial action units of these expressions and the relatively large inter-frame motion amplitude. The stable visual cues reduce the complexity of feature extraction. In contrast, for low-confidence emotions (such as neutrality and contempt), due to the small movement amplitude of facial muscles and the subtle feature differences, the recognition accuracies of traditional methods are generally lower than 80%. The MADV-Net method proposed in this paper achieves an average recognition accuracy of 89.53% based on the SAMM dataset [15], surpassing the existing state-of-the-art (SOTA) methods by 5.2 to 8.7 percentage points (the specific comparative data are shown in Table 5). It is worth noting that this algorithm maintains excellent performance for both high-confidence emotions and low-confidence samples. The macro-F1 value reaches 0.88, an improvement of 0.04 compared with the second-best method, demonstrating the stability and balance of cross-category recognition. MADV-Net has the ability to achieve dynamic feature modeling and can accurately capture the subtle motion differences between frames in micro-expression sequences. By effectively distinguishing between expressionless states and pseudo-neutral expressions, it avoids the misjudgment problems caused by traditional methods that rely on single-frame features. For the strong feature signals of high-confidence emotions, MADV-Net uses a spatial attention module to strengthen the feature extraction of key regions (such as the mouth area and the eyebrows), suppressing background noise interference. In the face of the weak signal features of low-confidence samples, the temporal attention mechanism dynamically allocates frame-level weights, highlighting the feature contributions of peak expression frames. This adaptive attention mechanism enables the algorithm to achieve the optimal feature combination in different emotion categories with a feature signal-to-noise ratio difference of 3:1, reducing the intra-class confusion error.

### 4.4. Quantitative Result Analysis

#### 4.4.1. Experimental Results on SMIC Dataset

**Figure 3:**
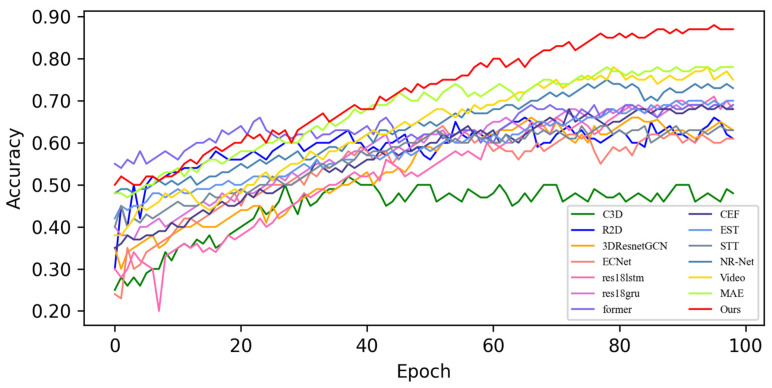
Average accuracy of the SOTA algorithms compared with the experimental results based on the SMIC dataset.

**Figure 4:**
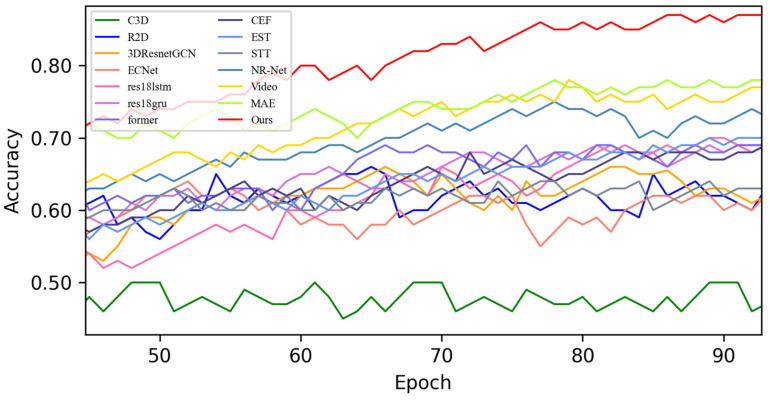
Enlarged view of the average accuracy of the SOTA algorithms compared with the experimental results based on the SMIC dataset.

In this section, based on MADV-Net and 13 SOTA methods, the average recognition accuracy and loss function value in the training process are compared based on the SMIC [12] dataset. Figure 3 shows the changes in average emotion recognition accuracy of all the comparative algorithms in the first 100 epoch experiments. Different algorithms are represented by curves with different colors. For the specific correspondence, refer to the legend in the upper left corner of the figure. The accuracy (red) of MADV-Net proposed in this paper for emotional sample recognition continues to rise steadily after 60 epochs, and the recognition accuracy curve is obviously higher than that of the other algorithms. Figure 4 shows that the model converges quickly after 60 epochs and effectively learns the expression correlation in the video stream, and the subsequent recognition accuracy curve is obviously higher than that of the other algorithms. To sum up, it is proven that the MADV-Net method proposed in this paper has better emotion recognition performance.

**Figure 5:**
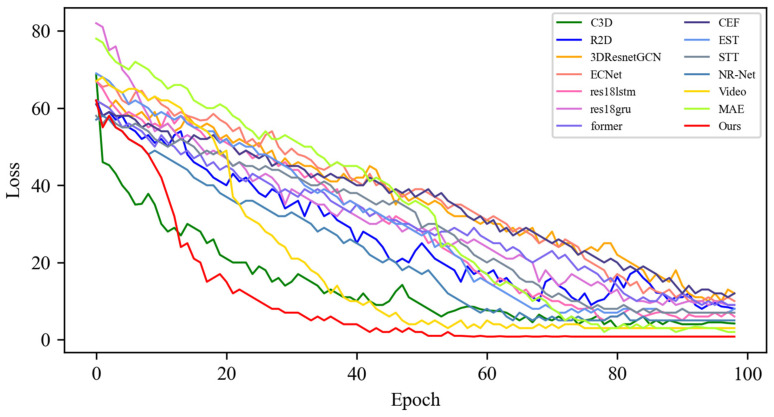
Experimental results of the loss value based on the SMIC dataset.

**Figure 6:**
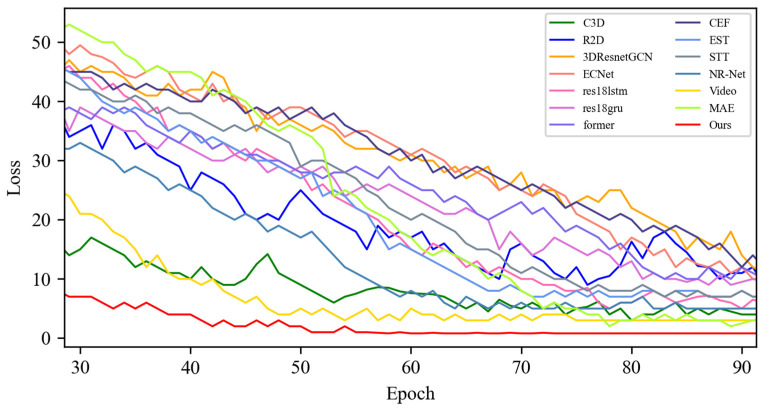
Local enlargement of the loss value results based on the SMIC dataset.

Figure 5 shows the changes in the loss values of all the compared algorithms in the first 100 epochs, and Figure 6 provides an enlarged view of some of the details of the loss changes. Through the analysis, it can be seen that after 20 epochs, the loss function of MADV-Net decreases rapidly, showing the best performance. Although C3D [36] showed a low loss value before 20 epochs, its subsequent loss value decreased gradually, and the final stable loss value was significantly higher than that of MADV-Net proposed in this paper. To sum up, the algorithm proposed in this paper shows good emotion recognition performance and stable prediction ability based on the SMIC [12] dataset.

#### 4.4.2. Experimental Results on CASME-II Dataset

**Figure 7:**
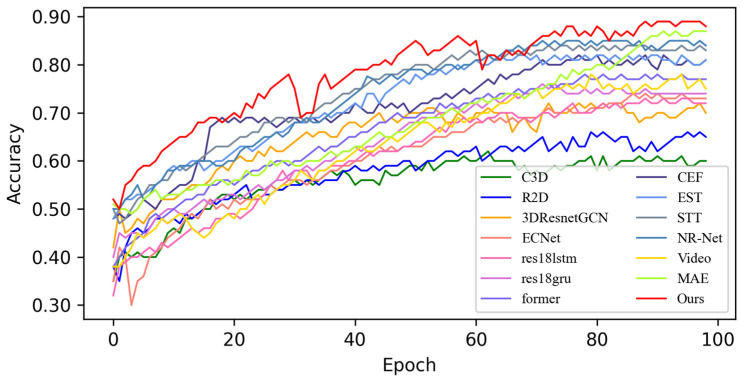
Average accuracy of the SOTA algorithms compared with the experimental results based on the CASME-II dataset.

**Figure 8:**
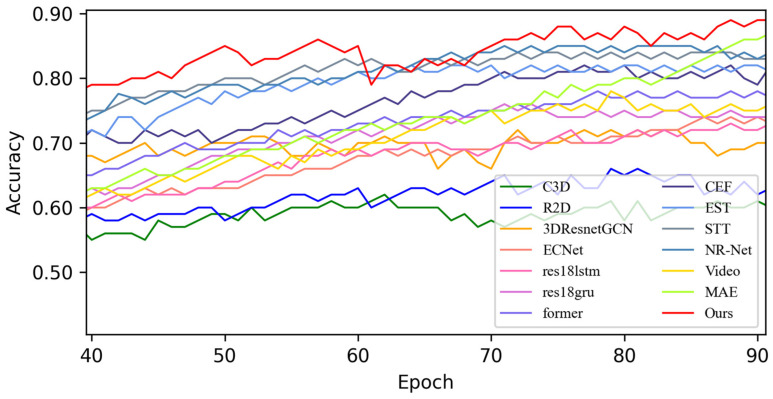
Enlarged view of the average accuracy of the SOTA algorithms compared with the experimental results based on the CASME-II dataset.

Figure 7 shows the quantitative results of the average recognition accuracy of MADV-Net and 13 SOTA methods based on the CASME-II [13] dataset. As can be seen from the figure, the classical C3D [37] and R2D [38] methods have similar performance, and the average recognition performance is low, meaning that it cannot meet the requirements of micro-expression emotion recognition. EST [44] and STT [45] rely on the powerful generalization ability of Transformer, and both of them have achieved stable accuracy of emotion recognition and a good ability to learn features. Although the average recognition accuracy of the recently proposed MAE [48] is lower than that of the other SOTA methods in the first 80 epochs, the average recognition accuracy after 80–100 epochs rises rapidly and finally reaches 86.50%. Compared with the above-mentioned SOTA methods, the MADV-Net method proposed in this paper achieved better average recognition accuracy and can complete the fine-grained classification of micro-expressions in CASME-II [13] data samples. The red line represents the results for MADV-Net, showing a rapid upward trend in the first 20 epochs, which proves that the network model can quickly extract the fine-grained features of different micro-expressions in the samples after initialization. After 80 epochs, it gradually converges and stabilizes, and the final average recognition accuracy reaches 89.50%, which is better than that of the other 13 SOTA methods for quantitative comparison. For the convenience of observation, the local average recognition accuracy of 40–100 epochs is enlarged in Figure 8.

**Figure 9:**
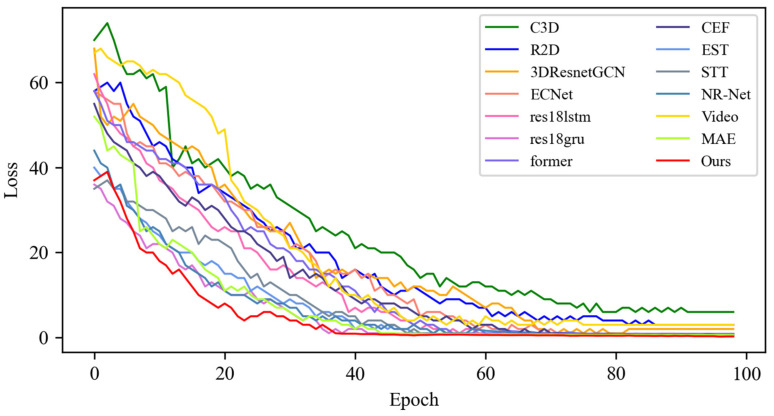
Experimental results of the loss value based on the CASME II dataset.

**Figure 10:**
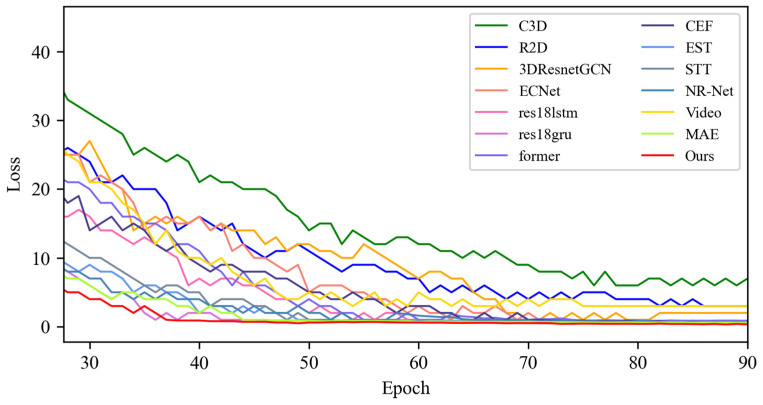
Local enlargement of the loss value results based on the CASME II dataset.

Figure 9 shows the quantitative results of the loss values of the SOTA methods and MADV-Net based on the CASME-II [13] dataset. Through quantitative loss analysis, it can be seen that the lower the loss value, the better the performance of the algorithm. The loss function of MADV-Net proposed in this paper and the 13 SOTA methods compared in the first 40 epoch decreases rapidly, and its decreasing trend reflects the ability of the algorithm to extract fine-grained features from large samples in the CASME-II [13] dataset. The faster the loss value decreases, the faster the algorithm can learn within-class features for different emotions. The red line represents the MADV-Net method proposed in this paper. Its decline rate is faster than that of the SOTA method, and the stationary loss value after 40 epoch is at a low point. For the convenience of observation, the variation in the local key loss values is presented in an enlarged view in Figure 10. Combined with the analysis of the average recognition accuracy curve, this confirms that MADV-Net has a higher average recognition accuracy and lower loss than the SOTA method based on the CASME-II [13] dataset.

#### 4.4.3. Experimental Results on CAS(ME)^2^ Dataset

**Figure 11:**
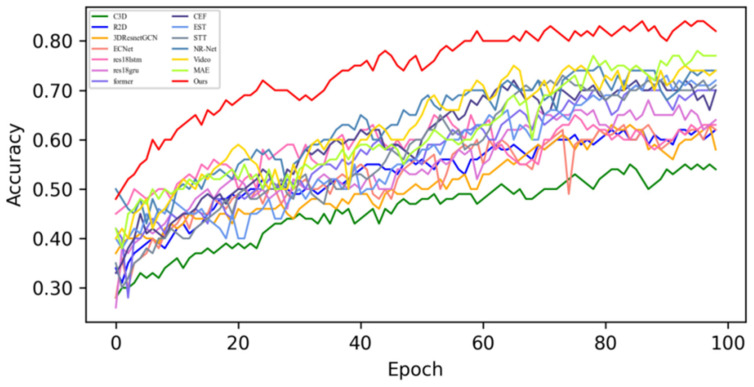
Average accuracy of the SOTA algorithms compared with the experimental results based on the CAS(ME)^2^ dataset.

Figure 11 shows the quantitative results of the average recognition accuracy of MADV-Net and the 13 SOTA methods proposed in this paper based on the CAS(ME)^2^ [14] dataset.

**Figure 12:**
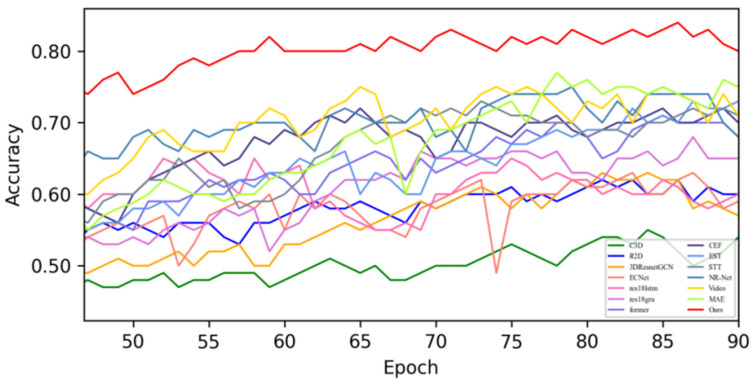
Enlarged view of the average accuracy of the SOTA algorithms compared with the experimental results based on the CAS(ME)^2^ dataset.

Because the CAS(ME)^2^ [14] dataset has a resolution of 200 fps and the facial area reaches a high facial resolution of about 280 × 340 pixels, MADV-Net can accurately capture the subtle dynamic changes in micro-expressions. At the same time, seven kinds of emotions in the dataset are marked with AUs, which provides support for MADV-Net to analyze more accurate intra-class and inter-class feature information through facial motion coding units. For the convenience of observation and differentiation, Figure 12 shows the local amplification results. The final verification shows that the MADV-Net method proposed in this paper is 20% higher than the other SOTA methods in recognition accuracy.

**Figure 13:**
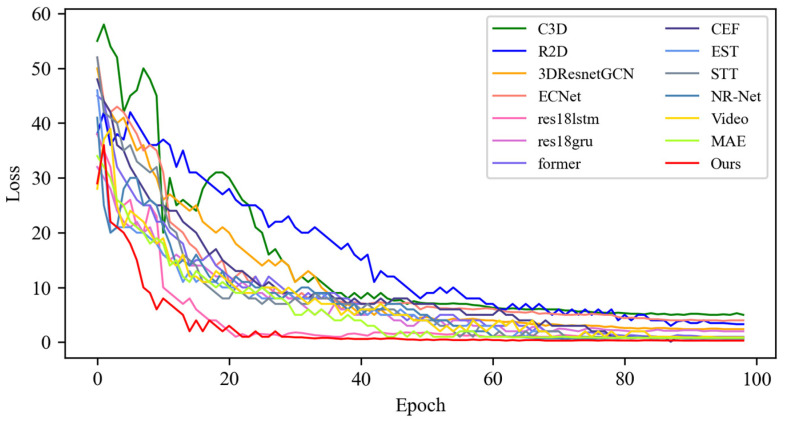
Experimental results of the loss value based on the CAS(ME)^2^ dataset.

Figure 13 presents the quantification results of the loss values of MADV-Net and other SOTA methods based on the CAS(ME)^2^ [14] dataset. The analysis shows that the loss function value of MADV-Net drops rapidly at the initial stage of training, which reflects the model’s efficient learning ability for fine-grained features of micro-expressions.

**Figure 14:**
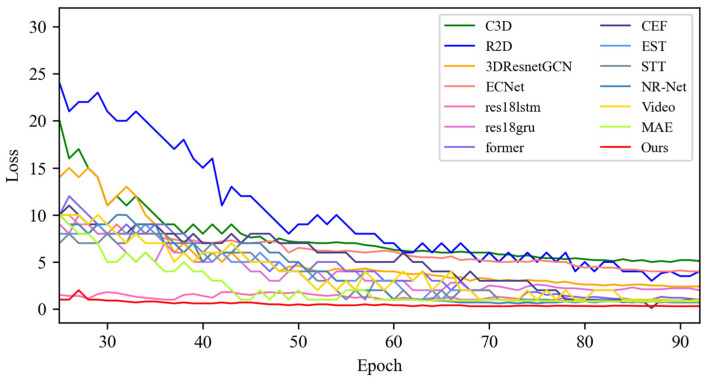
Local enlargement of the loss value results based on the CAS(ME)^2^ dataset.

The local amplification results in Figure 14 shows that MADV-Net approaches the minimum point of the loss function around the 30th epoch. This phenomenon further verifies that the model structure based on the current data samples can effectively guide the model to locate the downward direction of the loss function and realize the efficient promotion of the training process.

#### 4.4.4. Experimental Results on SAMM Dataset

**Figure 15:**
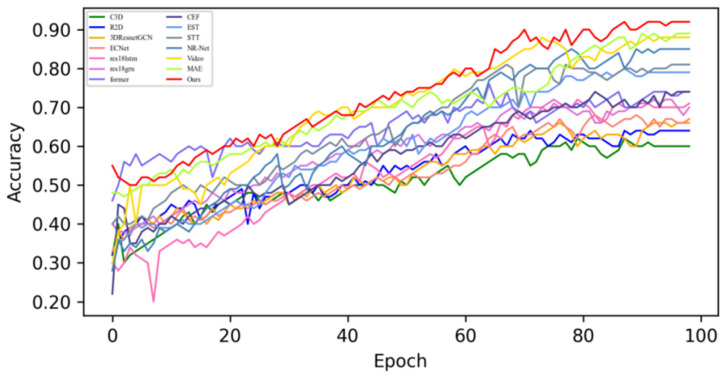
Average accuracy of the SOTA algorithms compared with the experimental results based on the SAMM dataset.

Figure 15 shows the experimental results for average accuracy based on the SAMM [15] dataset. The analysis shows that all the algorithms converge quickly in the initial stage of the model (0–40 epochs)—that is, the inter-class feature changes in video samples are quickly learned. Among them, the accuracy of this algorithm, VideoMAE [47], and MAE [48] is the most remarkable, and all three algorithms adopt a fine-grained attention design.

**Figure 16:**
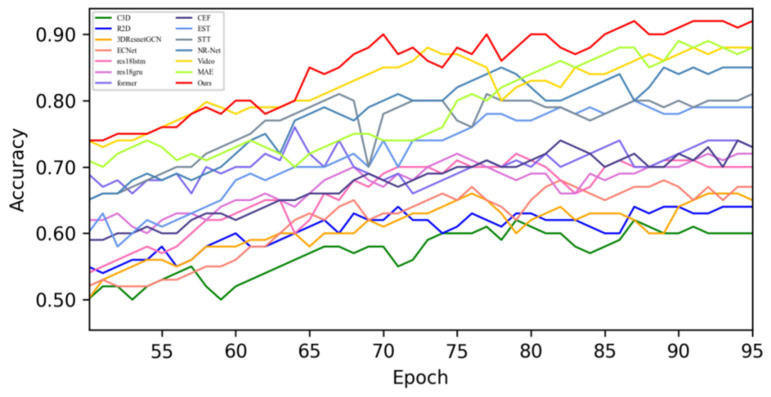
Enlarged view of the average accuracy of the SOTA algorithms compared with the experimental results based on the SAMM dataset.

For the convenience of observation, Figure 16 enlarges the local results of the experiment. The results show that at the critical stage of 50–100 epochs, the difference in average recognition accuracy of different algorithms gradually widens. VideoMAE [47] and MAE [48] have outstanding recognition rates, reaching 85% and 88%, respectively. EST [44] and STT [45] also achieved high accuracy, which proves that the methods based on the Transformer architecture have strong intra-class analysis ability and fast learning performance of inter-class features in dynamic and fast-changing recognition tasks such as micro-expressions. In this paper, based on the Transformer architecture, more abundant 3D depth information is further introduced, which is most prominent in the convergence stage of the algorithm, and finally, the average recognition accuracy is over 90%.

**Figure 17:**
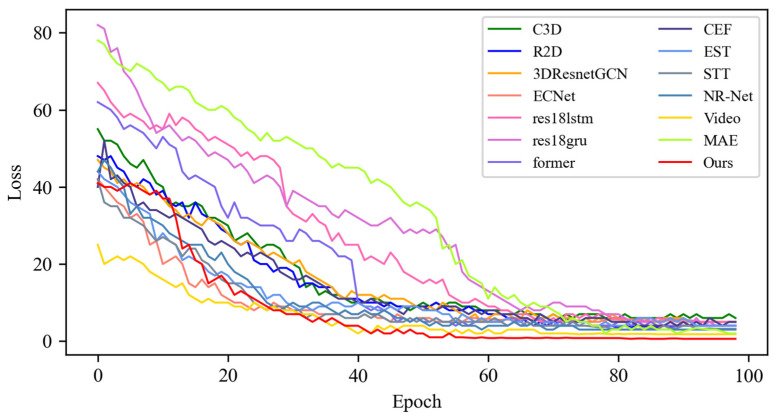
Experimental results of the loss values based on the SAMM dataset.

**Figure 18:**
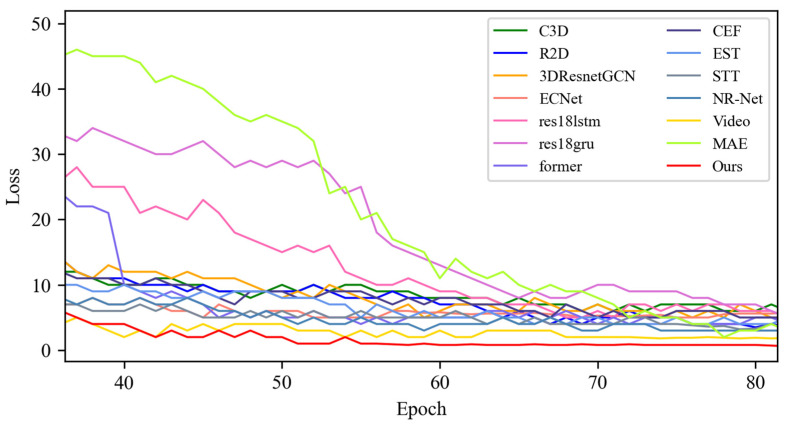
Local enlargement of the loss value results based on the SAMM dataset.

Figure 17 shows the changes in loss function values of different algorithms based on the SAMM [15] dataset. It can be seen from the figure that in the first 20 epochs, the loss function values of Resnet + LSTM [40], Resnet + GRU [41], Former [42], STT [45], EST [44], and the MADV-Net method proposed in this paper all decreased rapidly, and these network models were all improved based on the Transformer architecture, which confirmed their micro-expression recognition ability. Combined with the local enlargement in Figure 18, it can be seen that after 40 epochs, the loss value of MADV-Net proposed in this paper is obviously lower than that of the other SOTA methods and gradually tends to be stable. To sum up, based on the SAMM [15] dataset, MADV-Net has a higher micro-expression recognition ability and more stable robustness.

### 4.5. Visualization Experiment

**Figure 19:**
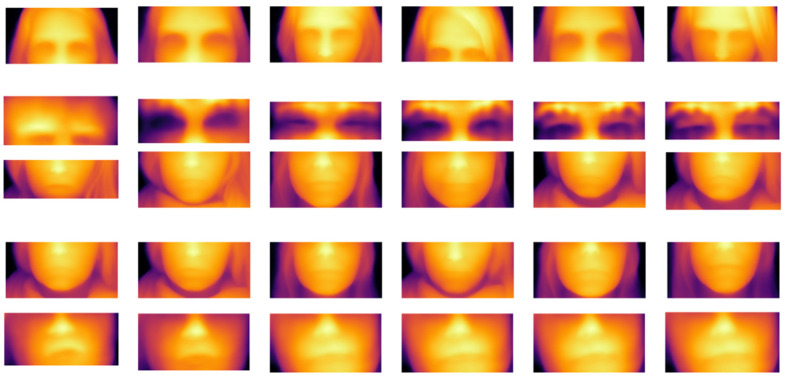
Depth disparity map of different AU regions of face.

The video streams of the depth parallax in different AU regions of the face from the AU data sample are visually displayed, and the results are shown in Figure 19. Depth disparity maps are presented for key facial AU regions, where the horizontal (x) and vertical (y) axes denote pixel coordinates in the facial ROI and color saturation represents depth values (z) encoding 3D muscle movement amplitude (see Section 3.2.1 for input processing). The analysis shows that the micro-expression dynamics in the eye area present a significant change feature in the depth disparity map: the greater the exercise intensity, the higher the color saturation corresponding to the depth value, which directly reflects the amplitude of the difference in muscle movement. However, the angle change of the mouth can be presented more clearly in the three-dimensional depth video stream, and its trajectory and angle difference are significantly enhanced by the depth information. Compared with traditional two-dimensional images, the three-dimensional depth representation can capture the subtle geometric deformation of the mouth more accurately. The visualization results verify the unique advantages of the depth parallax video stream in analyzing the dynamic characteristics of different AU regions of the face.

These depth maps correspond to the 3D depth stream input defined in Section 3.1 and are processed by the 3D-video-to-tokens module (Equation (2)), where each pixel’s z-value represents its distance from the camera. The intensity variations reflect the AU intensity differences modeled in Equation (3), demonstrating how MADV-Net leverages 3D geometry to enhance micro-expression feature learning.

Based on the MADV-Net method proposed in this paper, the following visual confusion experiments are carried out on the SMIC [12] dataset, CASME-II [13] dataset, CAS(ME)^2^ [14] dataset, and SAMM [15] dataset.

**Figure 20:**
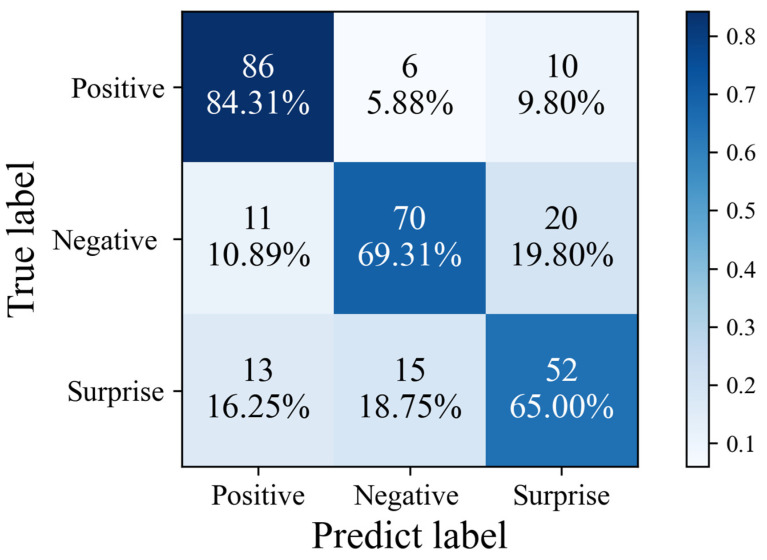
Confusion matrix diagram of the algorithm proposed in this paper based on the SMIC dataset.

As shown in Figure 20, among the samples of positive emotions in SMIC [12], 84.31% (86) were correctly predicted, only 5.88% were misjudged as negative emotions, and 9.80% were misjudged as surprise, so the recognition accuracy was relatively good. Among the negative emotion samples, 69.31% (70 samples) were correctly classified, 10.89% were misjudged as positive emotions, and 19.80% as surprise, meaning the misjudgment was concentrated in the surprise category. Among the surprise samples, only 65.00% (52 samples) were correctly identified, 16.25% were misjudged as positive emotions, and 18.75% were misjudged as negative emotions, indicating that this is the most difficult category to identify among the three categories, reflecting that the model has insufficient ability to capture its characteristics. To sum up, the model has the best recognition effect on positive emotions, but the ability to distinguish surprises is weak, and there are cross-category misjudgments in all categories, especially surprises, and negative emotions are easily confused.

**Figure 21:**
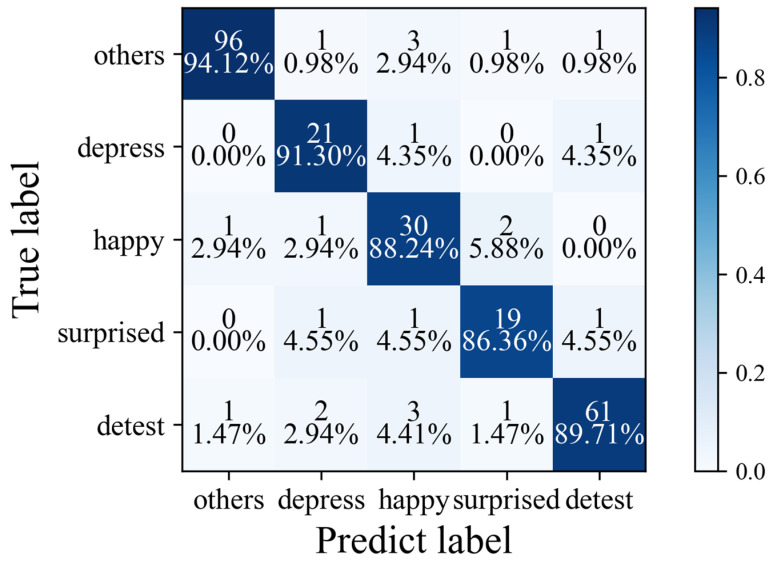
Confusion matrix diagram of the algorithm proposed in this paper based on the CASME II dataset.

Figure 21 shows the confusion matrix of the model based on CASME-II [13]. Among the depression samples, 91.50% (21 samples) were correctly classified, and only 4.35% were misjudged as happy, so the model has a strong ability to distinguish this category. For happy emotions, 88.24% (30) correctly predicted, and some were misjudged as other emotions (2.94%) or surprise (5.88%), which led to the confusion of category characteristics. The cause of misjudgment is the visual overlap of facial muscle movements of some emotions. For example, happiness and surprise may have subtle similarities in the dynamic characteristics of facial expressions (such as the muscle stretching range and facial features’ deformation mode), which makes it difficult for the model to accurately distinguish the differences between classifications.

**Figure 22:**
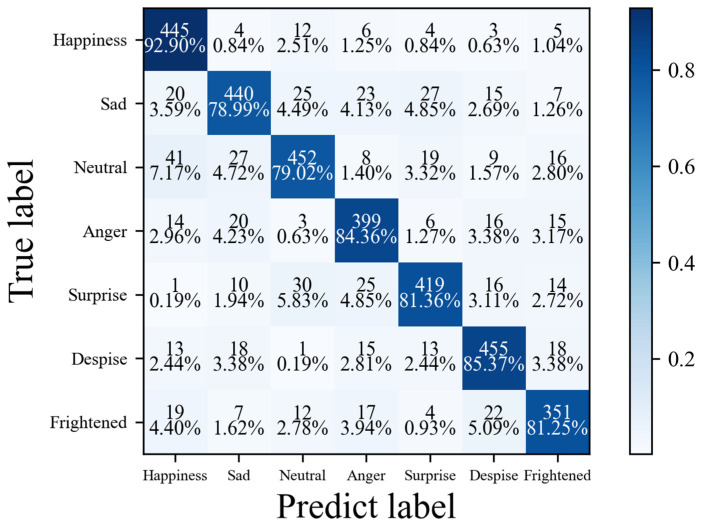
Confusion matrix diagram of the algorithm proposed in this paper based on the CAS(ME)^2^ dataset.

Figure 22 shows the confusion matrix of the model based on CAS(ME)^2^ [14]. Among the emotions, the highest recognition accuracy is observed for happy emotions, at 92.90%, while some instances are misjudged as neutral emotions (2.51%). The correct recognition rates of disgust and fear are 85.37% (455) and 81.25% (351), respectively. There are certain similarities between and within the two types of samples, which makes the model prone to misrecognizing the above emotions. It is also possible that there are subjective differences between the emotional samples themselves, and the labels of the two types of samples are blurred, which causes the training data to carry invisible noise and interfere with the model discrimination. Comprehensive analysis shows that MADV-Net has stable recognition accuracy for the seven kinds of emotions, and it also has good generalization for samples with high similarity within the class.

**Figure 23:**
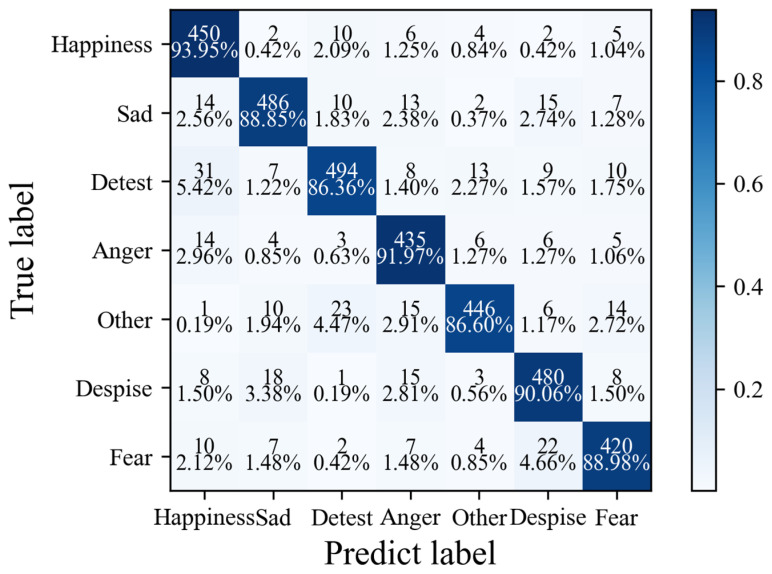
Confusion matrix diagram of the algorithm proposed in this paper based on the SAMM dataset.

Figure 23 shows the confusion matrix of the model based on SAMM [15]. Due to the abundant samples and complete label construction of this dataset, the average accuracy rate of MADV-Net in the recognition of seven kinds of emotions is higher than 86%, among which the intra-class characteristics of happy and angry samples are obvious, so the recognition accuracy rate is as high as 93.95% and 91.97%, respectively. Due to the introduction of an AU-based encoder module in MADV-Net, the correct recognition rate of 86.36% (494 samples) can still be achieved for samples indicating hatred with weak regularity. It is proven that the algorithm proposed in this paper has efficient discrimination ability and strong generalization for small samples or samples with unclear feature distribution between classes.

## 5. Conclusions

The adaptive dynamic visual attention model for micro-expression recognition proposed in this paper solves the problems of difficulty in capturing dynamic micro-expression features in video streams and low recognition accuracy. Differing from the traditional transformer encoder model, the model proposed in this paper consists of mixed attention, factorial self-attention, and dot product self-attention based on AU coding, which extracts the feature information in the depth space dimension, time dimension, and face AU dimension, respectively, and improve the learning ability of micro-expression features in video streams through adaptive learning strategies. After that, the maximum likelihood function of AU facial coding is introduced to minimize the loss value, and Sigmoid is used to regularize the compression parameters in the training process to improve convergence speed. Finally, the function is used for optimization. Based on SMIC [12], CASME-II [13], CAS(ME)^2^ [14], and SAMM [15], the average recognition accuracy of micro-expressions is 72.87%, 89.94%, 83.32%, and 89.53%, respectively. These values are higher than those for the mainstream micro-expression recognition and analysis algorithms. It is proven that the algorithm proposed in this paper has the ability to perform high-precision dynamic identification and high-speed parameter calculation.

## Figures and Tables

**Table 1:** AUs for different emotions. Adapted from [28], with permission from publisher.

Emotion	Action Units	FACS Name
Happiness	6 + 12	Check raiser Lip corner puller
Sadness	1 + 4 + 15	Inner brow raiser Brow lowerer Lip corner depressor
Surprise	1 + 2 + 26 + 5B	Inner brow raiser Outer brow raiser Slight Upper lid raiser Jaw drop
Fear	1 + 2+4 + 5+7 + 20 + 26	Inner brow raiser Outer brow raiser Brow lowerer Upper lid raiser Lid tightener Lip stretcher Jaw drop
Anger	4 + 5+7 + 23	Brow lowerer Upper lid raiser Lid tightener Lip tightener
Disgust	9 + 15 + 16	Nose wrinkler Lip corner depressor Lower lip depressor
Contempt	R12A + R14A	Lip corner puller (right side) Dimpler (right side)

**Table 2:** Comparative experimental results of different algorithms on SMIC.

Method	Positive Emotion Recognition Accuracy (%)	Accuracy of Negative Emotion Recognition (%)	Surprise Recognition Accuracy (%)	Average Recognition Accuracy (%)	Macro-F1
C3D [37]	58.15	40.83	45.00	47.99	0.44
R(2 + 1)D- 18 [38]	61.86	49.04	48.26	53.05	0.50
3D ResNet-18 [39]	62.67	45.87	47.35	51.96	0.49
EC-STFL [40]	61.06	44.68	46.15	50.63	0.50
Resnet-18 + LSTM [41]	68.03	52.13	51.24	57.13	0.53
Resnet-18 + GRU [42]	66.54	54.21	52.00	57.58	0.55
Former-DFER [43]	67.68	54.79	56.43	59.63	0.56
CEFLNet [44]	67.67	51.67	52.08	57.14	0.56
EST [45]	70.10	55.67	52.87	59.54	0.57
STT [46]	62.14	60.09	53.20	58.47	0.56
NR-DFERNet [47]	73.49	58.31	62.60	64.80	0.60
VideoMAE [48]	75.19	58.41	63.50	65.70	0.61
MAE-DFER [49]	76.02	65.70	61.25	67.65	0.62
**MADV-Net**	**84.31**	**69.31**	**65.00**	**72.87**	**0.69**

**Table 3:** Comparative experimental results of different algorithms on CASME-II.

Method	Happy (%)	Constrain (%)	Surprised (%)	Detest (%)	Other (%)	Average Recognition Accuracy (%)	Macro-F1
C3D [37]	54.00	62.25	49.25	66.00	68.50	60.00	0.56
R(2 + 1)D- 18 [38]	62.30	66.70	58.60	67.20	68.60	64.68	0.59
3D ResNet-18 [39]	65.00	68.00	72.00	71.50	71.50	69.60	0.61
EC-STFL [40]	66.25	68.68	74.00	72.50	74.00	71.08	0.68
Resnet-18 + LSTM [41]	74.00	71.50	70.50	74.20	75.20	73.08	0.69
Resnet-18 + GRU [42]	74.50	76.00	74.50	74.00	70.20	73.84	0.68
Former-DFER [43]	78.00	78.00	77.60	75.50	77.50	77.32	0.71
CEFLNet [44]	79.50	81.20	80.00	81.50	84.00	81.24	0.76
EST [45]	86.00	79.60	82.00	80.00	81.60	81.84	0.78
STT [46]	86.50	81.25	82.45	80.60	82.00	82.56	0.79
NR-DFERNet [47]	86.50	85.00	86.00	84.50	82.50	84.90	0.80
VideoMAE [48]	87.00	88.20	79.50	85.50	85.00	85.04	0.81
MAE-DFER [49]	90.00	86.50	86.00	84.00	86.00	86.50	0.82
**MADV-Net**	**94.12**	**91.30**	**88.24**	**86.36**	**89.71**	**89.94**	**0.84**

**Table 4:** Comparative experimental results of different algorithms in CAS(ME)^2^.

Method	Happy (%)	Sad (%)	Neutral (%)	Angry (%)	Surprised (%)	Despise (%)	Frightened (%)	Average Recognition Accuracy (%)	Macro-F1
C3D [37]	48.20	45.53	52.71	53.72	63.45	54.93	60.23	54.11	0.52
R(2 + 1)D-18 [38]	79.65	39.02	56.65	51.02	67.25	63.25	62.08	59.84	0.55
3D ResNet-18 [39]	76.32	50.20	64.15	61.95	46.53	61.02	62.65	60.40	0.58
EC-STFL [40]	78.25	50.05	54.25	60.25	65.25	62.83	60.57	61.63	0.59
Resnet-18 + LSTM [41]	81.90	60.95	62.60	66.97	53.25	60.20	60.82	63.81	0.60
Resnet-18 + GRU [42]	80.65	61.50	61.45	68.51	52.02	70.89	71.54	66.65	0.62
Former-DFER [43]	83.58	67.58	67.00	70.00	56.25	73.54	71.57	69.93	0.64
CEFLNet [44]	84.24	64.56	67.01	70.03	52.00	80.00	81.00	71.26	0.66
EST [45]	86.25	65.25	67.15	72.54	78.81	65.21	79.25	73.49	0.68
STT [46]	87.12	64.25	62.65	71.56	63.21	73.48	75.68	71.13	0.69
NR-DFERNet [47]	88.15	64.25	68.95	69.58	60.51	81.56	82.15	73.59	0.70
VideoMAE [48]	91.25	68.15	70.52	74.02	61.56	85.61	79.65	75.82	0.71
MAE-DFER [49]	91.85	70.95	72.56	75.21	65.21	80.56	83.69	77.14	0.72
**MADV-Net**	**92.90**	**78.99**	**79.02**	**84.36**	**81.36**	**85.37**	**81.25**	**83.32**	**0.78**

**Table 5:** Comparative experimental results of different algorithms based on SAMM.

Method	Happy (%)	Sad (%)	Detest (%)	Angry (%)	Other (%)	Despise (%)	Frightened (%)	Surprised (%)	Average Recognition Accuracy (%)	Macro-F1
C3D [37]	65.00	57.00	58.60	60.00	62.00	61.20	68.00	61.64	61.68	0.58
R(2 + 1)D- 18 [38]	67.50	60.00	63.20	67.00	61.00	64.00	68.50	64.40	64.45	0.59
3D ResNet-18 [39]	68.00	69.00	72.00	70.00	61.00	63.20	64.00	66.72	66.74	0.61
EC-STFL [40]	72.00	70.00	68.00	68.00	64.00	65.00	65.00	67.36	67.42	0.63
Resnet-18 + LSTM [41]	74.50	74.00	70.00	72.00	74.00	68.50	68.00	71.56	71.57	0.65
Resnet-18 + GRU [42]	78.00	75.00	67.50	68.00	65.00	74.00	70.00	71.06	71.07	0.66
Former-DFER [43]	80.00	82.00	70.00	72.00	74.00	72.00	73.00	74.68	74.71	0.68
CEFLNet [44]	80.00	78.00	72.00	78.00	70.00	71.00	73.50	74.62	74.64	0.69
EST [45]	82.00	82.00	85.00	81.00	75.00	74.50	76.50	79.36	79.42	0.71
STT [46]	84.00	82.50	86.00	84.00	80.00	76.00	78.00	81.50	81.50	0.79
NR-DFERNet [47]	86.00	86.00	85.00	86.00	85.00	86.00	84.00	85.36	85.42	0.81
VideoMAE [48]	92.00	94.00	90.00	86.00	84.00	85.00	86.00	88.12	88.14	0.82
MAE-DFER [49]	91.00	94.00	92.00	88.00	94.00	86.00	84.00	89.80	89.85	0.84
**MADV-Net**	**93.95**	**88.85**	**86.36**	**91.97**	**86.60**	**90.06**	**88.98**	**89.47**	**89.53**	**0.88**

## Data Availability

The datasets used in the experiment are all from open-source datasets. SMIC: http://www.cse.oulu.fi/SMICDatabase (accessed on 15 May 2025); CASME-II: http://casme.psych.ac.cn/casme/c2 (accessed on 15 May 2025); CAS(ME)^2^: http://casme.psych.ac.cn/casme/c3 (accessed on 15 May 2025); SAMM: http://www2.docm.mmu.ac.uk/STAFF/M.Yap/dataset.php (accessed on 15 May 2025).

## Abbreviations

The following abbreviations are used in this manuscript:

MaE	Macro expression
ME	Micro expression
FER	Facial expression recognition
SFER	Static facial expression recognition
DFER	Dynamic facial expression recognition
AU	Action unit
MADV-Net	Multi-level adaptive dynamic visual attention network model
FACS	Facial Motion Coding System
